# Insights Into Coronary Artery Lesions in Kawasaki Disease

**DOI:** 10.3389/fped.2020.00493

**Published:** 2020-08-25

**Authors:** Danfeng Zhang, Lingjuan Liu, Xupei Huang, Jie Tian

**Affiliations:** ^1^Ministry of Education Key Laboratory of Child Development and Disorders, Department of Cardiology, National Clinical Research Center for Child Health and Disorders, China International Science and Technology Cooperation Base of Child Development and Critical Disorders, Children's Hospital of Chongqing Medical University, Chongqing, China; ^2^Chongqing Key Laboratory of Pediatrics, Chongqing, China; ^3^Department of Biomedical Science, Charles E. Schmidt College of Medicine, Florida Atlantic University, Boca Raton, FL, United States

**Keywords:** Kawasaki disease, coronary artery lesions, genetic susceptibility, biomarker, long-term outcomes

## Abstract

This review summarizes recent advances in understanding the development of coronary arteritis in Kawasaki disease. Kawasaki disease is the most common cause of acquired heart disease among children characterized with coronary artery abnormalities, which can cause myocardial ischemia, infarction, and even death. The pathogenic factors of Kawasaki disease and the pathological process of coronary artery disease are not clear at present, which brings challenges to the prevention and treatment of the disease. The treatment of Kawasaki disease focuses mainly on timely administration of intravenous high doses of immunoglobulin and aspirin. However, there are still some patients who do not respond well to this standard treatment, and its management remains a challenge. As a result, coronary artery lesions still occur in patients and affect their quality of life. In this review, we discuss updated research data of Kawasaki disease coronary artery lesions.

## Introduction

The first case of Kawasaki disease (KD) was described in the 1960s, by Tomisaku Kawasaki (he termed it mucocutaneous lymph node syndrome then, and now it is called Kawasaki disease), which is an acute febrile illness, self-limited of unknown etiology, which mainly affects children under 5 years of age, especially those under 3 years of age (1). Coronary artery lesions in Kawasaki disease patients are not initially detected until cardiac complications have been observed in 1–2% of patients. Most of the sudden deaths result from coronary artery occlusion and rupture in patients (6/37) (2–4). A long-term prognosis is measured by the severity of coronary artery lesions. Some patients have a risk of coronary thrombosis and stenosis, leading to ischemia and even sudden death. As a result, Kawasaki disease is now the leading cause of acquired heart disease in developed countries, not rheumatic heart disease (5, 6). Coronary artery lesions have been reduced from 25 to 5% after treatment with intravenous immunoglobulin (IVIG) plus aspirin (7). However, due to different levels of diagnosis and treatment in different regions, treatment delay (i.e., longer than 10 days after fever) may occur, and some patients fail to respond to conventional treatment regimens. These patients have an increased chance of severe coronary artery damage that causes death or requires a heart transplant. Fortunately, randomized controlled clinical trials of IVIG plus steroid or infliximab in primary treatment have shown a significant reduction of this risk (8, 9). Coronary artery is the most frequently involved site in Kawasaki disease (10). The main manifestations include destruction of vessel wall structure, dilation, rupture, stenosis, and even obstruction of the vessels. This review focuses on recent progress related to the understanding of CAL in Kawasaki disease.

## Epidemiology of CAL

The degree of CAL varies from simple dilation to different size and number of aneurysms during the acute phase and even to stenosis or occlusion of the lumen most likely involving the left main coronary artery followed by the right coronary artery in a Chinese study (11).

When intravenous immunoglobulin (IVIG) combined with aspirin is used in the treatment of Kawasaki disease, the incidence rate of CAL decreases significantly. Currently, after initial therapy with IVIG combined with aspirin, coronary artery lesions mainly occur in patients who do not respond to IVIG therapy and are in complete Kawasaki disease (may be due to the delay of treatment) (12, 13).

However, there are regional differences in CAL incidence rates. From 2004 to 2014 in Canada, CAL affected 3.5% of all patients (14). In the United States, 2.25–3.20% of patients with Kawasaki disease suffered from coronary artery aneurysms, but higher in the West (27.1%) (*Z* score) (15, 16). In the United Kingdom, a study shows, from 2013 to 2015, the CAL rate was 19% in all patients (*Z* score) (17). In Australia, a recent study shows the incidence of coronary artery (CA) dilatation is 16.7%, and 6.8% had CA aneurysms based on absolute diameter measurements of coronary arteries (18).

Several studies show a highest incidence among Asian and Pacific islanders (19). In Taiwan, from 1976 to 2007, CAL (based on absolute diameter measurements) were about 20.2–31.5% in Kawasaki patients (20). In Korea, 12.66% of Kawasaki patients showed CAL based on absolute diameter measurements (21). In Shanghai from 2008 through 2012, 15.9% of Kawasaki cases developed CAL defined as dilation or aneurysm (based on absolute diameter measurements) (11). In Japan, a nationwide survey showed about 9.7% of Kawasaki patients experienced acute-phase CAL (based on absolute diameter measurements), and 2.8% experienced CAL in the follow-up procedure (1 month after onset) (coronary dilatation, 1.8%; giant aneurysms, 0.18%; coronary stenosis, 0.02%, and myocardial infarction, 0.004%). Coronary artery lesion varies with gender and age. Men and infants are more prone to coronary artery lesion (22).

In general, the rate of coronary artery lesion in Kawasaki disease varies with different regions and races even in the same region at different times (20), which is probably related to local environment, different diagnostic criteria, and other pathological factors. However, the pathological agents are not identified so far.

## Pathophysiology of CAL

A sequential model of Kawasaki disease vascular lesions was proposed in the early autopsy results of children who died of Kawasaki disease. This model suggests that neutrophils infiltrate the coronary arteries during the first 1–2 weeks of the disease, and then the neutrophils are replaced by monocytes, and the inflammation subsides automatically within 2 months after fever onset (23). The inflammatory process is manifested as endothelial dysfunction, the destruction of collagen and elastic fibers, and the loss of the structural and functional integrity of damaged coronary arteries, leading to a thickening of the intima, the interruption of laminar flow and blood flow, and thrombosis. However, this does not explain the presence of chronic vascular inflammation in a small number of patients who die months after the onset and subsequent endothelial dysfunction and thickening of the intima (24–26). In 2012, Rowley research group performed a large-sample pathology study that identified necrotizing arteritis, subacute chronic arteritis, and luminal myofibroblast proliferation—three interrelated rather than progressive pathologic processes in Kawasaki disease CAL pathological changes (27). Necrotizing arteritis occurs within 2 weeks of onset and is usually a self-limiting process characterized by infiltration of neutrophils in the vascular wall that begins when endothelial cells are stimulated by inflammatory factors in serum and express adhesion molecules and receptors on the surface of endothelial cells that leads to progressive necrosis of endothelial cells, mediators, and the outer membrane of medium arteries, especially coronary arteries. Subacute chronic arteritis is the infiltration of lymphocytes/macrophages, plasma cells, and eosinophils and can be found in all cases. Takahashi also describes that the inflammatory cells that appear in the coronary arterial lesions are mainly composed of macrophages in all patients. In addition, numerous neutrophils are also identified in the coronary arterial lesions of the patients who died 10 days after the onset of KD (28). However, in some cases (small number), the earliest time is 6 days after the onset, indicating a lack of time continuity. The earliest infiltrating cells cannot be identified. Inflammatory activation of the coronary artery depends on multiple inflammatory pathways, especially pathways associated with activated T lymphocyte function and type I interferon-induced pathways (29). The MMPS family, especially mmp-2 and mmp-9, can cause the destruction of middle structures of the arteries resulting from the degradation associated with CAL (30–32). Subacute chronic vasculitis also occurs in the first 2 weeks and is accompanied by infiltration of other inflammatory cells, involving blood vessels throughout the body, but mainly involving medium-sized arteries, especially coronary arteries. Myofibroblast proliferation is closely related to subacute chronic vasculitis and is a unique process involving the proliferation of myofibroblasts and the accumulation of matrix degradation products that gradually block the arterial lumen. The origin of these myofibroblast-like cells is unknown; one research finds endothelial-mesenchymal transition (EndoMT) can be activated by sera from KD patients, but there is no further study on whether this is related to myofibroblast-like cells (33). Current research in other models suggest that they may originate from multiple sources, including vascular smooth muscle cells by losing differentiation marker smoothelin, perivascular progenitor cells via proliferation, vascular endothelial cells (ECs) through EndoMT, and circulating or adventitial fibroblasts via epithelial-mesenchymal transition (EMT) (34–38). Whether these mechanisms are the same in Kawasaki disease needs further study. The failure to restore normal coronary artery lesions may be due to the persistence of vessel wall inflammation and coronary artery endothelial dysfunction.

These pathological processes are based on autopsy of dead patients or heart transplant patients, which are the most severe cases and do not fully reflect the whole process of Kawasaki disease coronary artery lesions.

## Biomarkers for Diagnosis of CAL

At the beginning, echocardiography was not widely used to evaluate coronary artery lesions in patients. Clinicians proposed different scoring systems to evaluate coronary artery lesions in patients with Kawasaki disease according to their characteristics, blood test results, and clinical course. Asai and Kusakawa's scoring system was widely used in the 1970s and 1980s, followed by Harada's in the 1990s. However, the sensitivity and specificity of this score vary from region to region. Currently, the evaluation of coronary artery lesions in Kawasaki disease mainly relies on color Doppler ultrasound, but ultrasound cannot accurately reflect the specific situation of coronary artery damage (39). It is reported that patients with normal ultrasound can still have coronary artery endothelial dysfunction, intima, and media change (40). Accurate diagnosis of coronary artery lesions is the focus of our attention. Biomarkers are necessary to assist in the determination of coronary artery lesions. There are some potential biomarkers of CAL formation (Table 1). Plasma clusterin level, NT-proBNP, CRP, and IL-6 are highly suggestive of coronary artery lesions (41, 43, 44). Increased expression of nitric oxide synthase (iNOS) in neutrophils suggests the occurrence of coronary artery lesions in KD patients (42). Kenichi et al. report higher serum sLR11 level may be the biomarker of CAL at the convalescent phase (45). Pi et al. find that 11-DH-TXB2, sP-selectin, IPF, and sCD40L levels are related to the degree of CAL (48). MicroRNA (miRNA) is considered to be one of the most promising biomarker resources in various types of nucleic acid research, including KD (49, 50). Xing et al. and Li et al. show that high levels of mir-92a-3p and miR-182-5p are a high risk factor for the occurrence of CAL (46, 47).

**Table 1:** Biomarkers associated with CAL formation.

**Biomarker**	**Clusterin**	**iNOS**	**Il-6**	**CRP**	**NT-proBNP**	**sLR11**	**mir-92a-3p**	**miR-182-5p**	**11-DH-TXB2, sP-selectin, IPF, sCD40L**
No. of cases	14	24	22	22	197	23	12	11	21
No. of control	33	31	50	50	664	20	18	50	23
Ethnicity	Taiwanese	Japaneses	Indian	Indian	Asian	Japanese	Chinese	Chinese	Chinese
Sensitivity (%)	69.7	NA	81.8	72.7	0.82	NA	81.8	NA	NA
Specificity (%)	64.3	NA	82	74	0.72	NA	66.7	NA	NA
Diagnostic criteria	Absolute diameter	Absolute diameter	Z score	Z score	Absolute diameter and Z score	Absolute diameter	Absolute diameter	Z score	Absolute diameter
Level of evidence	3B	3B	3B	3B	3A	3B	3B	3B	3B
References	Yu et al. (41)	Yu et al. (42)	Nandi and Pal (43)	Nandi and Pal (43)	Zheng et al. (44)	Watanabe et al. (45)	Rong et al. (46)	Li et al. (47)	Pi et al. (48)

Other studies show that matrix metalloproteinases degrade the extracellular matrix, leading to matrix remodeling. The imbalance of the MMP family is also one of the markers of CAL, especially MMP-9 and MMP-9:MMP-2 (32, 51). Liu et al., using a protein general analysis, found five significantly differentially expressed proteins in patients with CAL, including kininogen 1 (KNG1), complement factor H (CFH), fibronectin 1 (FN1), mannose binding lectin 2 (MBL2), and serpin family C member 1 (SERPINC1). However, these are not specific markers for CAL (52).

A take-home message is that, although variety of biological markers have emerged, no specific biological markers so far are confirmed for the diagnosis and prognosis of the disease because of the lack of multicenter and multispecies clinical data. Although the detection of NT-proBNP and CRP is highly feasible compared to other clinical assays, the sensitivity and specificity are limited.

## Genetic Background of CAL

Kawasaki disease is a disease closely related to genetic susceptibility. However, it is not clear whether there is a correlation between coronary artery lesions and genetic polymorphism.

Based on several genome-wide association studies (GWAS) and other gene polymorphism studies, the data suggest that CAL formation in KD is related to various genetic factors, such as HLA-E, HLA-B, CD40, FCGR2A, HMGB1, MICB, PELI1, ITPKC, CASP3, MMP (MMP-3, MMP-13), IL-10, ITPR3, and so on (Table 2) (53–59, 61, 63–68). Onouchi et al. identify a functional SNP (rs28493229) in the ITPKC gene on chromosome 19q13.2 that may be significantly correlated with the progression of CAL through the Ca2+/NFAT signaling pathway as a negative regulator of T-cell activation but only in Japanese and Caucasian populations. CD40 can activate both humoral and cellular immunity by stimulating antigen-presenting cells and vascular endothelial cells. Kuo et al. indicate that the genetic polymorphisms of CD40 (rs4810485) are involved in CAL of KD in a Taiwanese population. A Korean GWAS study showed that the functional SNP (rs17136627) of a KCNN2 gene that plays a role in potassium mediators/small conductance calcium activation channels in KD patients without CAL and in patients with medium or giant aneurysms is closely related to the development of severe CAL (70). Kuo et al. find that gene combinations [LOC100133214 (rs2517892) and IL2RA(rs3118470)] influence CAL in 384 SNPs (71). The TGF-βsignaling pathway may play an important role in EMT and pro-inflammatory cell infiltration in CAL formation (72). Shimizu et al., using a candidate gene approach, find variants in genes in the transforming growth factor (TGF)-β signaling pathway (TGFβ2, TGFβR2, and SMAD3) are associated with the *Z* scores of CAL in patients of Euro-Americans and Koreans (69, 73).

**Table 2:** Genes associated with CAL formation.

**Gene**	**Chromosome Location**	**Method**	**Populations**	**References**	**Potential mechanism**
HLA-B HLA-E	6p21.3	Genotyping Meta-analysis	Chinese, Taiwanese	Lin et al. (53) Xie and Shi (54) Hsieh et al. (55)	Antigen-presentation
ITPKC	19q13.2	Genotyping	Japanese, Taiwanese	Onouchi et al. (56) Kuo et al. (57)	C calcineurin/NFAT pathway
CD40	20q13	Genotyping	Taiwanese	Kuo et al. (58)	Immune activation
HMGB1	13q12.3	Genotyping	Koreans	Ahn et al. (59)	Gene transcription
MICB	6p21.3	TaqMan Allelic Discrimination assay	Taiwanese	Hsieh et al. (60)	Antigen-presentation
CASP3	4q34-35	Genotyping, Two-locus gene model	Japanese, Taiwanese	Onouchi et al. (61) Kuo et al. (57)	Gene transcription
KCNN2	5q22.3	GWAS	Koreans	Kim et al. (62)	Potassium mediators/small conductance calcium activation channels
FCGR2A	1q23.3	Genotyping	Japanese	Taniuchi et al. (63)	Signal transmission
Il-10	1q32.1	Genotyping	Han Chinese, Taiwanese, Koreans	Lin et al. (64)	NA
ITPR3	6p21.3	Genotyping	Taiwanese	Huang et al. (65)	innate immune responses
MMP	11q22.2	Genotyping	Japanese, Euro-Americans, Koreans	Ikeda et al. (66) Shimizu et al. (67) Park and Shin (68)	Inflammation and Tissue remodeling
TGF-βR2	3p24.1	Direct sequencing	Koreans	Choi et al. (69)	TGF-β pathway
PELI1	2p13.3	GWAS	Koreans, Taiwanese	Kim et al., (62)	TLR signaling

Although there are lots of genomic-based studies, the limitation of these studies is obvious because these studies receive data based on local population, small sample size, and different definitions of CAL. Furthermore, how to relate these genomic data with phenotype analyses is still a challenge. More studies with different populations are needed in the future.

## Immune Mechanisms of CAL

Kawasaki disease is an inflammation of blood vessels throughout the body. Abnormal activation of the immune system is an important link in the development of coronary artery lesions.

The innate immune system (cellular and humoral) is involved in the disease in an early stage characterized by the production of numerous neutrophils, majority γδT cells, pathogen-associated molecular patterns (PAMPs), the elevated levels of damage-associated molecular patterns (DAMPs), and circulating inflammatory cytokines, such as interleukin (IL) 1, IL-6, and tumor necrosis factor -α (TNF-α) in the acute stage (74–76). Meanwhile, the intestinal mucosal permeability and sIgA are closely related to vasculitis in a KD animal model (77). A CAWS-induced model indicates that NLRP3 in BMDCs are important for vasculitis formation (78). The markers associated with antigen presentation (CD74, CD1c, CD20, TLR7) and activated myeloid dendritic cells are significantly elevated in coronary tissue (79, 80).

The activation of the adaptive immune system appears in the later stage of the disease, mainly as the increase of regulatory T-cells, and memory T- and memory B-cells. The self-limited nature of the disease coupled with a low rate of recurrence and the presence of oligo-clonal IgA implies that Kawasaki disease is associated with a super-antigen rather than with a conventional antigen. In the animal model of Kawasaki disease, vasculitis induced by LCWE, CD8+ T-cells, rather than CD4+ T-cells, NK T-cells, or TReg cells, is an important factor in the production of CAL (81). Interestingly, in patients with CAL, CD40L was highly expressed in CD4+ T-cells and platelets although there was no significant difference between CD8+ T-cells and serum (82). As a distinct subset of CD4+ T-cells, Tfh 1 (follicular T helper) cells in the CALs+ group were significantly lower. In contrast, cTfh2 cells in the CALs+ group significantly increased (83).

The immunological responses vary with different pathological conditions. It is understandable that the different studies mentioned above produced different results, which are probably due to the different animal models used and the different disease stages applied in the studies. As for why the immune response is focused on the vascular wall in KD, no studies have been proposed so far, which may be related to the vascularity of related antigens.

## Diagnosis and Treatment of CAL

The American Heart Association scientific statement in 2004 and Japanese guidelines in 2008 classify abnormality using *Z* score (≥2.5) but aneurysms by absolute dimensions (6, 84). AHA classified aneurysms based on *Z* scores until 2017 (CAl: *Z* score > 2 or a decrease in *Z* score ≥ 1 during follow-up) (85). There are seven systems to calculate *Z* score; these systems differ by age range, race, the formula used to calculate BSA, and the regression method used for analysis. Canadian subjects by Dallaire and Japanese subjects by Kobayashi are more rigorous than others (86, 87). The *Z-*score is better than the absolute dimensions in assessing the severity of coronary artery dilatation in proximal segments (88), but the left circumflex branch is not available. Because of the limitation of ultrasound, computed-tomographic angiography, cardiac magnetic resonance imaging, and invasive angiography can be used when necessary.

Currently, the main method of KD therapy is intravenous immunoglobulin (IVIG) combined with aspirin, which can effectively reduce systemic inflammatory response but has no direct effect on endothelial cells. Based on the understanding of genetic influences on CAL susceptibility, A clinical trial in Japan has found cyclosporine, which can block the calcineurin-NFAT pathway, can significantly reduce the incidence of CAL (12/86 patients vs. 27/87 patients) at higher risk for IVIG resistance (89). Avastatin can inhibit regular T-cell function, endothelial/epithelial mesenchymal transformation, and the abnormal expression of MMPS, and now, a Phase I/IIa clinical trial is currently underway (90). In addition, low-density lipoprotein can lower cholesterol, and the pleiotropic effects of statins in endothelial cells can improve endothelial cellular function, decreasing oxidative stress and alleviating inflammation (91). Infliximab (a TNFα receptor blocker) cannot reduce the incidence of CAL; however, it can effectively alleviate the progression of CAL during the follow-up (9, 92). In one study, prednisolone reduces the incidence of CAL in patients who are not responding to IVIG (3% vs. 23%) (8).

Although there have been some positive results (Table 3), existing experiments have been limited to a specific population, and further studies are needed to determine whether they are effective in other populations. In addition, how to accurately predict the occurrence of IVIG resistance and CAL is the premise of the use of these protocols since the use of additional treatment is unnecessary in low-risk patients.

**Table 3:** Drugs that can reduce the occurrence or severity of CAL plus with immunoglobulin.

**Drug**	**Study design**	**Populations**	**Sample size**	**References**	***P*-value**
Cyclosporine	Randomized controlled trial	Japanese	175	Hamada et al. (89)	*p* = 0.010
Avastatin	Prospective	Americans	NA	Tremoulet et al. (9)	Under way
Infliximab	Randomized controlled trial	Americans	196	Tremoulet et al. (9)	*P* = 0.045
Prednisolone	Randomized controlled trial	Japanese	248	Kobayashi et al. (8)	*P* < 0.0001

## Long-Term Outcomes

Kato et al. followed up 598 patients for 10 to 21 years. Most of the patients (55%) with small or medium-sized coronary artery aneurysms returned to normal luminal dimension 6 to 18 months later, but some patients developed stenosis and MI. Those without CAL did not show abnormalities in the ultrasound follow-up (93). A follow-up of more than 10 years of coronary angiography revealed that, although some small and medium aneurysms returned to normal size, there was still morphological and vascular dysfunction, and there was no vascular dysfunction during follow-up in patients without CAL in the acute phase (94). After the death from KD (with or without CAL in the acute phase), chronic inflammation can be found in coronary artery pathological sections. It has been reported that, in Kawasaki disease patients, long-term isotope nuclear imaging can show high metabolism in the coronary site (95). Non-invasive assessment of arterial structure and function in 60 patients at least 2 years after KD, 60 patients with or without CAL, presented a high risk of cardiovascular disease, including increased aortic IMT and carotid distensibility (96, 97). Other studies have shown that the segmental thickening of the intima can be found in the degraded coronary aneurysm after IVUS check, and even no CAL in the acute phase (98). Recent studies have reported new aneurysm onset or further expansion in the late period, and one patient without CAL died 3 years after the onset, probably due to coronary artery disease (99, 100). The prognosis of CAL mainly depends on the degree of stenosis of the lumen, but due to the limitations of coronary angiography and autopsy, the incidence of it in follow-up data is insufficient. In patients, intimal thickening with calcification in coronary aneurysm segment detected by intravascular ultrasound in Kawasaki disease outbreak a few years later are similar to adult coronary atherosclerosis change (101).

Coronary artery lesions are a chronic process, and it is necessary to have a careful follow-up study to observe patients because some vascular dysfunction can occur later even when coronary aneurysm disappears. In some cases, no CAL was found in the acute phase, which suggests, in general, a good prognosis. However, a long-term cardiovascular risk is still possible. It is difficult to make it clear because the follow-up data so far are limited to monitoring the middle age and aging patients with a high incidence of cardiovascular disease.

## Conclusion

Kawasaki disease was considered to be a self-limited disease in the past. However, according to the abovementioned information, we can find that coronary artery lesions in Kawasaki disease are a chronic process, and there are still abnormalities in the coronary artery structure or function during convalescence. For the treatment of Kawasaki disease, especially for the treatment of coronary artery lesions, more studies are needed on the mechanisms underlying the occurrence of coronary artery lesions, which can provide us with information regarding precise molecular targets of intervention. Follow-up is an important part of the CAL long-term outcome, which mainly relies on the echocardiogram. However, echocardiograph examination has its limitations that cannot accurately assess CAL. Thus, more specific and sensitive coronary artery lesion biomarkers and advanced imaging technology should be useful and helpful. So far, the long-term follow-up data are still scarce. Therefore, performing long-term follow-up observation of Kawasaki disease and collecting data from follow-up in the future is crucial for us to have a deeper understanding of this disease.

## Author Contributions

DZ contributed to conception and wrote the first draft of the manuscript. All authors contributed to manuscript revision, read, and approved the submitted version.

## Conflict of Interest

The authors declare that the research was conducted in the absence of any commercial or financial relationships that could be construed as a potential conflict of interest.
